# Effects of Fasting and Feeding on Transcriptional and Posttranscriptional Regulation of Insulin-Degrading Enzyme in Mice

**DOI:** 10.3390/cells10092446

**Published:** 2021-09-16

**Authors:** Carlos M. González-Casimiro, Patricia Cámara-Torres, Beatriz Merino, Sergio Diez-Hermano, Tamara Postigo-Casado, Malcolm A. Leissring, Irene Cózar-Castellano, Germán Perdomo

**Affiliations:** 1 Unidad de Excelencia Instituto de Biología y Genética Molecular, University of Valladolid-CSIC, 47003 Valladolid, Spain; carlosmanuel.gonzalez.casimiro@uva.es (C.M.G.-C.); patricia.camara@alumnos.uva.es (P.C.-T.); bmerino@ibgm.uva.es (B.M.); tamara.postigo@uva.es (T.P.-C.); irene.cozar@uva.es (I.C.-C.); 2 Institute for Research in Sustainable Forest Management (iuFOR), University of Valladolid, 34004 Palencia, Spain; sergio.diez.hermano@uva.es; 3 Institute for Memory Impairments and Neurological Disorders, University of California, Irvine (UCI MIND), Irvine, CA 92697-4545, USA; m.leissring@uci.edu; 4 Centro de Investigación Biomédica en Red de Diabetes y Enfermedades Metabólicas Asociadas (CIBERDEM), 28029 Madrid, Spain

**Keywords:** insulin-degrading enzyme, fasting, refeeding, insulin resistance, liver, metabolic adaptations, nutritional state, metabolic flexibility, lactate

## Abstract

Insulin-degrading enzyme (IDE) is a highly conserved and ubiquitously expressed Zn^2+^-metallopeptidase that regulates hepatic insulin sensitivity, albeit its regulation in response to the fasting-to-postprandial transition is poorly understood. In this work, we studied the regulation of IDE mRNA and protein levels as well as its proteolytic activity in the liver, skeletal muscle, and kidneys under fasting (18 h) and refeeding (30 min and 3 h) conditions, in mice fed a standard (SD) or high-fat (HFD) diets. In the liver of mice fed an HFD, fasting reduced IDE protein levels (~30%); whereas refeeding increased its activity (~45%) in both mice fed an SD and HFD. Likewise, IDE protein levels were reduced in the skeletal muscle (~30%) of mice fed an HFD during the fasting state. Circulating lactate concentrations directly correlated with hepatic IDE activity and protein levels. Of note, L-lactate in liver lysates augmented IDE activity in a dose-dependent manner. Additionally, IDE protein levels in liver and muscle tissues, but not its activity, inversely correlated (*R*^2^ = 0.3734 and 0.2951, respectively; *p* < 0.01) with a surrogate marker of insulin resistance (HOMA index). Finally, a multivariate analysis suggests that circulating insulin, glucose, non-esterified fatty acids, and lactate levels might be important in regulating IDE in liver and muscle tissues. Our results highlight that the nutritional regulation of IDE in liver and skeletal muscle is more complex than previously expected in mice, and that fasting/refeeding does not strongly influence the regulation of renal IDE.

## 1. Introduction

The global epidemics of obesity and type 2 diabetes mellitus (T2DM) are major public health concerns [1]. These overnutrition-related chronic diseases are associated with changes in diet and physical activity. Understanding physiological and pathophysiological metabolic adaptations to either fasting or food consumption are essential to preventing and treating these diseases, while minimizing the side effects caused by chronic overnutrition. However, the molecular and cellular mechanisms associated with metabolic adaptations to fasting and refeeding remain poorly understood.

Insulin-degrading enzyme (IDE) is a highly conserved metalloprotease ubiquitously expressed in insulin-responsive tissues, such as the liver, skeletal muscle and kidneys [2]. Although initially characterized as a protease of insulin more than 70 years ago by Mirsky and Broh-Kahn [3], it can degrade several other peptide substrates, including hormones (e.g., glucagon and somatostatin), amyloidogenic peptides (Aβ and amylin), chemokine ligands (CCL3 and CCL4) as well as oxidized hemoglobin and viral proteins [4]. Additionally, several non-proteolytic functions for IDE have been described involving a wide range of cellular targets such as receptors (e.g., insulin, androgen, and glucocorticoid), cytoskeletal components (e.g., vimentin, nestin, and α-synuclein), and cell-cycle regulators (e.g., retinoblastoma protein and tensin homolog deleted on chromosome 10 (PTEN)) [4]. Thus, IDE is a multifactorial protein with both proteolytic and non-proteolytic functions affecting multiple cellular processes of particular importance to the hormonal regulation of fasting (glucagon-mediated) and absorptive (insulin-mediated) states.

The impact of a high-caloric diet on IDE transcriptional and posttranscriptional regulation has been investigated in mice and rats [5,6,7,8,9,10,11,12]. However, in the published reports, the length and composition of diets, strains, and sex of animals are highly varied, precluding the drawing of definitive conclusions about the impact of feeding and diet composition on the regulation of IDE, particularly in multiple tissues. In Swiss male mice, for example, feeding a cafeteria diet for 8 weeks resulted in reduced *Ide* mRNA levels in liver and muscle tissues in parallel with lower IDE protein levels [6]. Similarly, C57BL/6 male mice fed a high-fat diet (HFD) for 12 weeks showed reduced hepatic IDE protein levels and activity [8,10], although muscle IDE protein levels and activity remained unchanged [8]. Interestingly, C57BL/6N mice fed an HFD (58% kcal in fat) for 12 weeks showed similar hepatic IDE protein levels but reduced activity [12]. Additionally, male C57BL/6J mice fed a high-fat/high-sugar diet for 16 weeks showed reduced hepatic protein IDE levels [11]. In contrast, C57BL/6J mice fed a high-fat diet (HFD; 58% kcal in fat) for 6 months caused higher hepatic IDE levels and activity, but lower *Ide* mRNA levels [7].

On the other hand, female Wistar rats fed a cafeteria diet for 17 weeks resulted in augmented hepatic *Ide* mRNA levels and activity [5]. Similarly, male Wistar rats fed a high-fructose diet (20% fructose) for 6 weeks showed lower hepatic *Ide* mRNA levels but higher hepatic IDE protein levels [9].

Studies in humans, revealed that serum IDE levels were higher in subjects with metabolic syndrome than in control subjects [13]. In African Americans (AAs), an ethnicity at higher risk for the development of T2DM and higher BMI compared with non-Hispanic whites (NHWs), hepatic IDE levels were similar compared with those of NHWs. However, hepatic IDE activity was lower in AAs compared to NHWs [14]. Consistently with these reports, subjects with T2DM showed reduced hepatic mRNA *Ide* levels [15].

In the present study, we performed an in-depth study of the regulation of *Ide* mRNA as well as IDE protein and activity levels in the liver, skeletal muscle, and kidneys of mice fed a standard and high-fat diet in response to the fasting-to-postprandial transition. Our studies uncovered the tissue-specific regulation of IDE mediated by hormones and metabolites.

## 2. Materials and Methods

### 2.1. Mouse Studies

Four- to five-week-old male C57BL/6J mice were purchased from Charles River Laboratories (Les Oncins, France). Mice were fed standard rodent diets (SD; Ssniff Spezialdiäten GmbH, Soest, Germany #V1534-703, 19% protein, 3.3% fat) or a high-fat diet (HFD; Research Diets #D12451, 35% carbohydrates, 45% fat) for 8 weeks. For the fasting-to-refeeding experiments, mice were fasted for 18 h. Subsequently, the group of mice fed an SD were refed with the same diet for 30 min or 3 h ad libitum. Likewise, the group fed an HFD were refed (30 min or 3 h) an HFD ad libitum. Caloric intake between mice refed an SD vs. HFD for 30 min were similar (3.2 ± 0.14 vs. 2.92 ± 0.35; *p* = 0.7). Likewise, the caloric intake between mice refed an SD vs. HFD for 3 h were also similar (1.4 ± 0.09 vs. 1.067 ± 0.09; *p* = 0.6). At the end of the fasting, 30 min refeeding, and 3 h refeeding period, mice were euthanized and blood collected. Liver, skeletal muscle (gastrocnemius), and kidneys were dissected and snap-frozen in liquid nitrogen. Tissues and blood samples were kept at −80 °C until analyses.

Mice were housed in ventilated cages under a 12:12 h light–dark cycle with water available ad libitum, at the animal facility of the University of Valladolid (UVa). The Animal Care and Use Committee of the UVa approved all experiments (protocol #5003931).

### 2.2. Plasma Biochemistry

Fasting and non-fasting (refeeding for 30 min or 3 h) blood glucose, and plasma triglycerides, cholesterol, insulin and glucagon levels were assessed as previously described [16,17]. Plasma non-esterified fatty acids (NEFAs), glycerol, and lactate levels were determined using the NEFA-HR (2) Assay Kit (Wako Chemicals, Neuss, Germany), L-Lactate Assay, and Glycerol Colorimetric Assay Kits (Cayman Chemical, Ann Arbor, MI, USA), respectively. The homeostatic model assessment (HOMA) index was calculated using fasting glucose and insulin concentrations according to Matthews et al. [18].

### 2.3. Quantitative Real-Time PCR

Total RNA was purified from the tissue samples of ~20 mg of mouse liver, kidneys and muscle, using TRIzol™ Reagent (Invitrogen™, Waltham, MA, USA) according to the manufacturer’s instructions. Quantification of total RNA was performed measuring ultraviolet absorbance in a NanoDrop^®^ ND-1000 full-spectrum spectrophotometer. The removal of any potential genomic DNA contamination was achieved by treating the samples with the RapidOut DNA Removal Kit (Thermo Scientific™, Waltham, MA, USA). Complementary DNA (cDNA) was synthesized using the iScript™ cDNA Synthesis Kit (Bio-Rad™, Madrid, Spain) according to the manufacturer’s instructions. The mRNA levels of target and housekeeping genes were determined by real-time quantitative PCR (qPCR) with TaqMan™ or SYBR™ Green assays on a LightCycler^®^ 480 System. qPCRs were carried out on equal cDNA amounts in triplicate for each sample, using the Maxima Probe qPCR Master Mix (Thermo Scientific™, Waltham, MA, USA) for TaqMan™ assays and Maxima SYBR Green qPCR Master Mix (Thermo Scientific™, Waltham, MA, USA) for SYBR™ Green assays. Data were analyzed with the two fit-points absolute quantification protocol, fixing the fluorescence threshold at 1.000. Target mRNAs’ expression levels were normalized using the 2^−ΔΔCt^ relative quantification method [19] normalized to the mRNA levels of the housekeeping gene, ribosomal protein L18 (*Rpl18*) using the following primers—forward: 5′-AAGACTGCCGTGGTTGTGG-3′; reverse: 5′-AGCCTTGAGGATGCGACTC-3′; probe: 5′-FAM-TTCCCAAGCTGAAGGTGTGTGCA-BHQ1–3′. TaqMan^®^ Gene Expression assay reference (from Applied Biosystems, Waltham, MA USA) for *Ide* was as follows: Mm00473077_m1. [16,17,20]. The following SYBR™ Green assays were used; *Ide*-15a—forward: 5′- CAGCCATGAGTAAGCTGTGG-3′; reverse: 5′- TCCCATAGATGGTATTTTGG-3′; *Ide*-15b: forward: 5′- CAGCCATGAGTAAGCTGTGG-3′ Reverse: 5′- TCAATAACCTGATAAACAGG-3′.

### 2.4. Western Blot Analyses

Western blot analyses on isolated mouse tissues were performed as previously described [16,17,20]. Briefly, aliquots (~20 mg) from liver, skeletal muscle (gastrocnemius), or kidneys were homogenized in 200 µL ice-cold lysis buffer (125 mmol/L Tris-HCl pH 6.8, 2% (*v*/*v*) SDS, 1 mmol/L dithiothreitol) supplemented with protease and phosphatase cocktail inhibitors (Sigma-Aldrich, St. Louis, MO, USA), and 1 mmol/l phenylmethylsulphonyl fluoride (PMSF; Merck Life Science, Darmstadt, Germany). Then, the lysates were sonicated for 3 min on ice, and centrifuged at 18,500× *g* for 10 min at 4 °C to separate and discard insoluble materials. Supernatants were kept and an aliquot was used for quantifying of the protein content using the Pierce BCA proteins assay kit (Thermo-Fisher, Waltham, MA, USA). Solubilized proteins (20 µg of muscle or kidney samples or 40 µg of liver extracts) were resolved by 10% SDS-PAGE and electrotransferred onto polyvinylidene difluoride (Immobilon-P, Merck-Millipore, Darmstadt, Germany) filters for immunoblotting by conventional means. After probing with a rabbit polyclonal anti-IDE antibody (1:40,000; #AB9210, Merck-Millipore, Darmstadt, Germany, USA), the membranes were stripped in buffer (2,5 mmol/L Tris-HCl pH 6.8, 2% (*w*/*v*) SDS, 0,7 % (*v*/*v*) β-mercaptoetanol) at 50 °C, and reprobed with antibody against actin for liver samples (1:40,000; #612656, BD Biosciences, Madrid, Spain) or tubulin for muscle and kidneys samples (1:5000; #2148S, Cell Signaling, Danvers, MA, USA). Signals were detected by chemiluminescence (Clarity Western ECL Substrate, Bio-Rad™, Madrid, Spain) and exposure to X-ray film to produce bands within the linear range. Band intensity was quantified with the ImageJ software (NIH, Bethesda, MA, USA). Briefly, developed films were scanned at 600 pixels per inches with HP Scanjet G4010 (Hewlett-Packard, Madrid, Spain) using the HP Photosmart Premier 6.5 software (Hewlett-Packard, Madrid, Spain). The obtained images (negative) were converted to a 32-bit format and were inverted in order to generate an image with detectable bands. Each band was individually selected with rectangular ROI selection, followed by the quantification of the peak area of obtained histograms. Actin or tubulin expression was determined to ensure similar protein loading. Data were acquired as arbitrary values.

### 2.5. IDE Activity

IDE activity was assessed with the fluorometric SensoLyte^®^ 520 IDE activity assay kit (AnaSpec, Inc., Fremont, CA, USA) according to the manufacturer’s instructions. The assay uses a unique FRET-based fluorogenic substrate derived from an amyloid precursor protein (APP) sequence designed to reduce cross reactivity with other peptidases such as neprilysin, disintegrin and metalloproteinase domain-containing protein 10 (ADAM10), tumor necrosis factor-α converting enzyme (TACE), beta-site amyloid precursor protein cleaving enzyme 1 (BACE-1), and BACE-2. The substrate also features a fluorophore that is excited at longer wavelengths than traditional fluorogenic substrates, making it less susceptible to interference by the autofluorescence of components in biological samples and test compounds. In the presence of the fluorogenic substrate, active IDE cleaves the substrate leading to an increase in 5-FAM fluorescence, which was monitored at excitation/emission = 490 nm/520 nm.

To prepare tissue extracts for activity assays, liver, muscle (gastrocnemius) or kidneys samples (~20 mg of tissue) were homogenized in 200 µL ice-cold assay buffer (AnaSpec, Inc., Fremont, CA, USA) in the presence of non-metalloprotease inhibitors (Protease Inhibitor Cocktail, Merck Life Science) plus 1mmol/L PMSF (Merck Life Science). Homogenates were incubated on ice for 15 min, followed by centrifugation at 10,000× *g* for 10 min at 4 °C to separate and discard insoluble materials. Supernatants (soluble tissue lysates) were kept, and an aliquot was used for quantifying the protein content using the Pierce BCA Protein Assay Kit (Thermo-Fisher, Waltham, MA, USA), yielding ~50–150 µg of total protein concentration per sample. Afterward, enzymatic reactions were set up by adding 50 µL of tissue lysates in a 96-well plate. The enzymatic reaction was started by adding 50 µL of fluorogenic substrate solution into each well. The plate was gently shaken for 30 s and sample fluorescence (5-FAM) was monitored on GENios Pro TECAN multiplate reader (TECAN, Männedorf, Switzerland) every 5 min for 100 min at 37 °C. Reactions were performed in duplicate per sample. As a positive control, purified recombinant human IDE (provided by the kit) was added to the reaction mix. As a negative control, equal amounts of tissue lysates from liver, muscle, or kidneys from IDE-KO mice were tested in parallel. Wells containing the reaction mix without tissue lysates were used as blanks to establish the background fluorescence levels, which were subtracted from all other readings from the same lysates. For kinetic analyses, all fluorescence readings were expressed in relative fluorescence units (RFU). RFU data were plotted versus time for each sample. Afterwards, we calculated the initial reaction velocity in RFU/min by determining the slope of the linear portion of the data plot. IDE specific activity is expressed as RFU/µg of total protein/min. The assay can detect as low as 0.8 ng/mL of active IDE.

To determine the effect of lactate on IDE activity, liver lysates or recombinant human IDE (provided by the SensoLyte^®^ 520 IDE activity assay kit) were assayed in the absence or the presence of sodium L-lactate (Sigma-Aldrich, St. Louis, MO, USA) at 2, 4, and 6 mmol/L.

### 2.6. Statistical Analyses

Statistical analysis was performed using Prism v. 6.0 (GraphPad Software, Inc., San Diego, CA, USA). The normality of the distribution of data was checked with the Kolmogorov–Smirnov test, then comparisons between two groups were assessed using the unpaired Student’s *t*-test. Data are presented as the means ± SEM. Comparisons between more than two groups were done using the one-way or two-way ANOVA followed by Bonferroni’s post hoc test for parametric data or the Kruskal–Wallis test for nonparametric data. Bivariate analyses were performed using the Pearson’s correlation coefficient. Differences were considered significant at *p* < 0.05.

Multivariate analysis was performed using R v.3.6.2 [21] and the R package *pls* v.2.7–3 [22]. Variables were centered and standardized prior to analysis to prevent biases due to different scales and dimensions. Principal component analysis (PCA) was performed through a partial least square (PLS) regression model fitted with the orthogonal scores algorithm [23]. Results were summarized in biplots showing the first two principal components and the combined effects of response variables alongside physiological variables. Graphics were plotted using the R packages *basicPlotteR* [24] and *plotrix* [25].

## 3. Results

### 3.1. Metabolic Responses to the Fasting-to-Refeeding Transition in Mice Fed an SD or HFD

**Figure A1:**
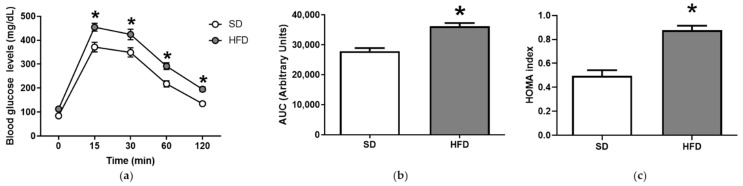
Mice fed an HFD exhibit impaired glucose homeostasis and insulin resistance. (**a**) Intraperitoneal glucose tolerance (IP-GTT) in mice fed an SD or HFD for 8 weeks. (**b**) Area under the curve (AUC) of the IP-GTT. (**c**) HOMA index. Data are the means ± SEM. N = 8 mice condition. * *p* < 0.05 vs. SD.

Mice fed an HFD exhibited glucose intolerance and insulin resistance (Appendix A, Figure A1), in parallel with an ~18% increased body weight (Table 1). Refeeding, measured 30 min and 3 h after refeeding augmented blood glucose and plasma insulin levels in mice fed an SD and HFD (Table 1). In response to 3 h refeeding, non-esterified fatty acids (NEFAs), and glycerol were reduced, but lactate levels were significantly increased in mice fed an SD (Table 1). In response to fasting, circulating glucagon levels were lower in mice fed an HFD compared to control mice (Table 1).

### 3.2. Liver IDE Expression and Activity

**Figure 1:**
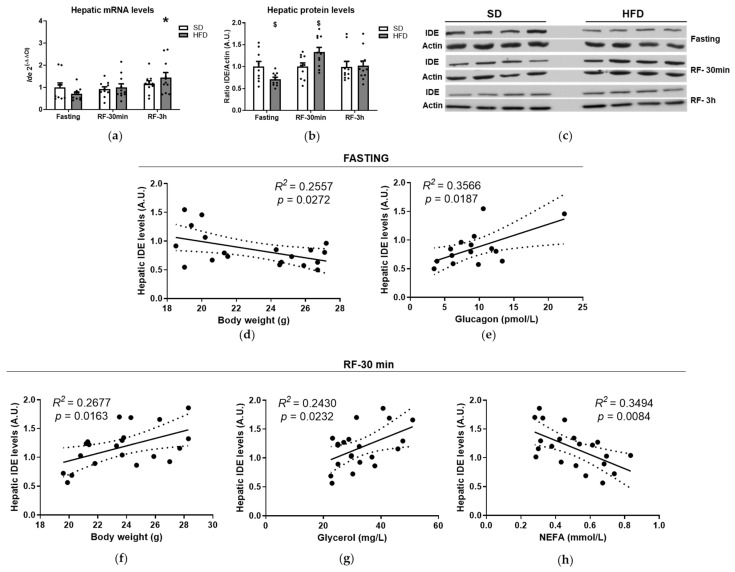
Transcriptional and posttranscriptional regulation of hepatic IDE. (**a**) Transcriptional regulation of *Ide*. Total mRNA was isolated and quantified from liver tissues of mice fed an SD or HFD during fasting or refeeding conditions. Data are the means ± SEM. N = 9–11 mice per condition. * *p* < 0.05 vs. fasting by ANOVA. (**b**) Regulation of IDE protein levels. Lysates from livers of mice fed an SD or HFD, during fasting or refeeding conditions, were resolved by Western blots and quantified by densitometric analyses using full blots (Appendix A, Figure A2a). Data were then plotted for each nutritional status and diet. Data are the means ± SEM. N = 9–11 mice per condition. ^$^ *p* < 0.05 vs. SD by ANOVA. (**c**) Representative image illustrating hepatic IDE levels showed in “panel b”. (**d**–**h**) Regression analyses. Bivariate analyses were performed using data from mice fed an SD and HFD during fasting or refeeding conditions. Correlations between fasting hepatic IDE protein levels and body weight (**d**), and glucagon (**e**). Correlations between hepatic IDE under refeeding (30 min) conditions and body weight (**f**), glycerol (**g**), and NEFA (**h**). The *R*^2^ and the statistical significances (*p*) values are indicated in the figure.

To investigate the impact of the fasting-to-refeeding transition on hepatic IDE regulation, C57BL/6J mice were fed an SD or HFD for 8 weeks. Neither fasting nor refeeding affected hepatic *Ide* gene expression in mice fed an SD. In contrast, *Ide* expression was upregulated (~2-fold) by refeeding (3 h) in mice fed an HFD (Figure 1a). No significant correlations were found between *Ide* mRNA expression levels and metabolic parameters in mice fed an HFD (Appendix A, Table A1). Although lactate and insulin levels showed a statistically significant correlation, the significance level was modest and the *R*^2^ value was rather low (Appendix A, Table A1). *Ide* expression levels also remained unchanged in mice fed an HFD compared to SD feeding (Figure 1a).

**Figure A2:**
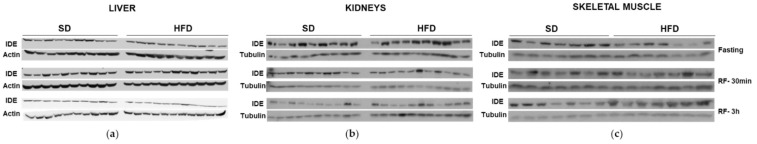
Effects of nutritional status on IDE protein levels. Mice were fed an SD or HFD for 8 weeks and IDE protein levels were analyzed under fasting and refeeding (30 min or 3 h) conditions. Full Western blot images for liver (**a**), kidneys (**b**), and skeletal muscle (**c**) are shown.

Of note, the transcriptional upregulation of *Ide* during refeeding (3 h) in mice fed an HFD was not paralleled with changes in protein levels (Appendix A, Figure A2a and Figure 1b,c). Neither fasting nor refeeding affected the hepatic IDE protein levels in mice fed an SD (Appendix A, Figure A2a).

With respect to IDE protein levels, fasting resulted in a significant reduction in hepatic IDE (~30%) in mice fed an HFD as compared to the mice fed an SD (Figure 1b,c). No correlations were found between IDE protein levels and metabolic parameters in fasting conditions (Appendix A, Table A2). Conversely, refeeding (30 min) upregulated IDE protein levels (~30%) in mice fed an HFD as compared to SD feeding (Figure 1b,c). Bivariate analysis revealed a direct correlation between hepatic IDE and circulating lactate levels (*R*^2^ = 0.4217, *p* = 0.03).

To further investigate the regulation of IDE protein levels, we performed bivariate analyses between IDE levels and various metabolic parameters in mice fed an SD and HFD. Under fasting conditions with hepatic IDE levels inversely correlated with body weight (Figure 1d) and directly correlated with plasma glucagon levels (Figure 1e). On the other hand, under refeeding (30 min) conditions, body weight and plasma glycerol concentration directly correlated with IDE levels (Figure 1f,g), whereas NEFA levels were inversely correlated (Figure 1h). These results indicate that changes in body weight, glucagon and metabolites (glycerol and NEFA) levels are able to predict hepatic IDE protein levels.

**Figure 2:**
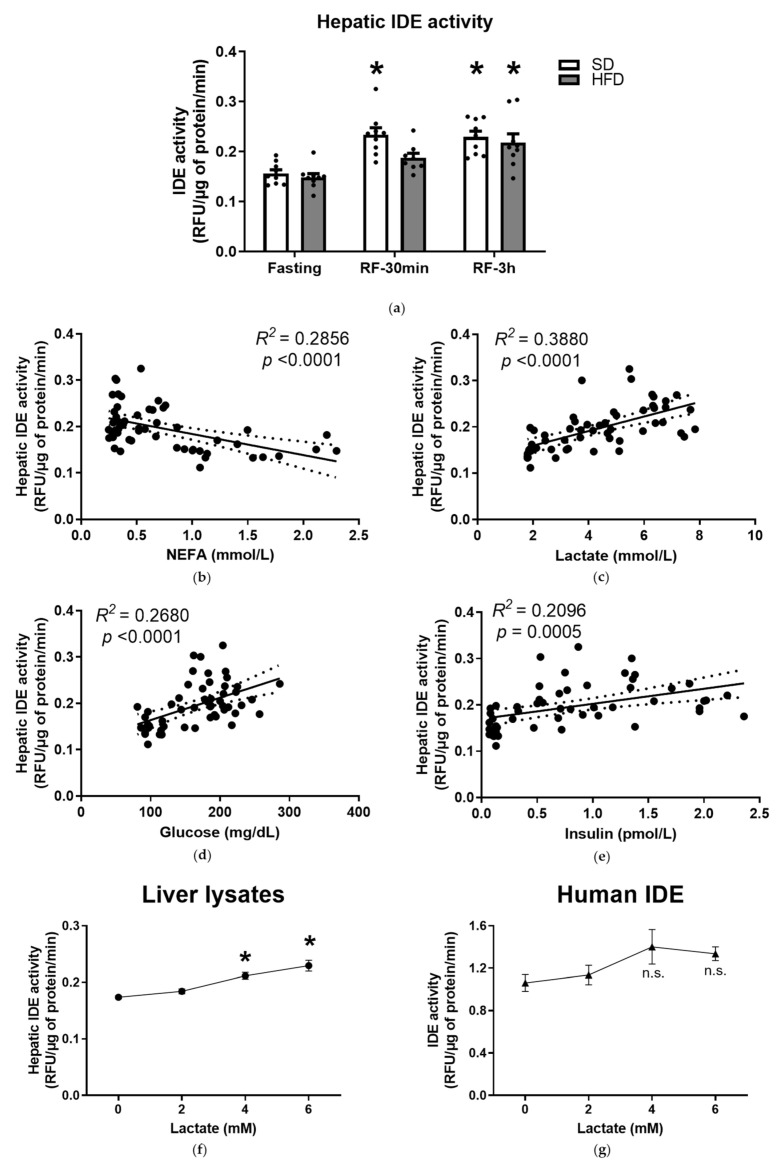
Effects of feeding and refeeding on hepatic IDE activity. (**a**) IDE activity in lysates from livers of mice fed an SD or HFD, during the indicated fasting or refeeding conditions. Data are the means ± SEM. N = 9 mice per condition. * *p* < 0.05 vs. (**b**–**e**) ANOVA and regression analyses between IDE activity in liver lysates and levels of different plasma biochemical parameters analytes. Bivariate analyses were performed on the data from mice fed an SD and HFD, during fasting or refeeding conditions. Correlations between hepatic IDE levels and NEFA (**b**), lactate (**c**), glucose (**d**), and insulin (**e**). The *R*^2^ and the statistical significances (*p*) values are indicated in the figure. (**f**–**g**) Effects of lactate on IDE activity. (**f**) IDE activity in the absence or presence of different concentrations of lactate in lysates from fasted livers of the group of mice fed an SD for 8 weeks. Data are the means ± SEM. N = 3 livers of mice per condition in duplicate. * *p* < 0.05 vs. no lactate by ANOVA. (**g**) Activity of purified human recombinant IDE in the absence or presence of different concentrations of lactate. Data are the means ± SEM. N = 3. n.s., not statistically significant.

In addition to gene expression and protein levels, we also investigated hepatic IDE activity in response to metabolic adaptations to nutritional changes. Refeeding increased IDE activity (~50%) in liver extracts from mice fed an SD (Figure 2a), which directly correlated with glucose (*R*^2^ = 0.4374, *p* < 0.001), lactate (*R*^2^ = 0.3583, *p* < 0.01), and insulin (*R*^2^ = 0.3711, *p* < 0.001); and inversely correlated with NEFA (*R*^2^ = 0.4778, *p* < 0.001) and glycerol (*R*^2^ = 0.3054, *p* < 0.01). Furthermore, refeeding also increased hepatic IDE activity (~50%) in mice fed an HFD (Figure 2a), which directly with lactate (*R*^2^ = 0.3861, *p* < 0.001); and inversely correlated with NEFA (*R*^2^ = 0.3748, *p* < 0.001). These results were similar to those obtained by the bivariate analyses of hepatic IDE activity in mice fed an SD and HFD, where IDE activity was inversely correlated with NEFA, but directly correlated with lactate, glucose and insulin levels (Figure 2b–e). Surprisingly, plasma lactate levels showed stronger correlation than circulating insulin (*R*^2^ = 0.388 vs. 0.2096) (Figure 2d,e).

As described above, we found a direct correlation between the circulating lactate levels and hepatic IDE activity. To further investigate whether this association was due to a causal relationship, we incubated the lysates obtained from fasted livers (of the group of mice fed an SD), or purified recombinant human IDE, with lactate levels that mimic those in circulation (Table 1). In a dose-dependent manner, lactate increased IDE activity (~20–30%) in liver lysates (Figure 2f), however, it had no significant effects on the activity of purified IDE (Figure 2g). Of note, the effect of lactate concentration on IDE activity levels observed in vitro (Figure 2f) is similar to that observed in liver extracts (Figure 2c). Taken together, these results demonstrate that lactate indirectly regulates hepatic IDE activity, lending support to the notion that the observed correlation between the circulating levels of lactate and hepatic IDE activity was due to a cause–effect relationship.

**Figure A3:**
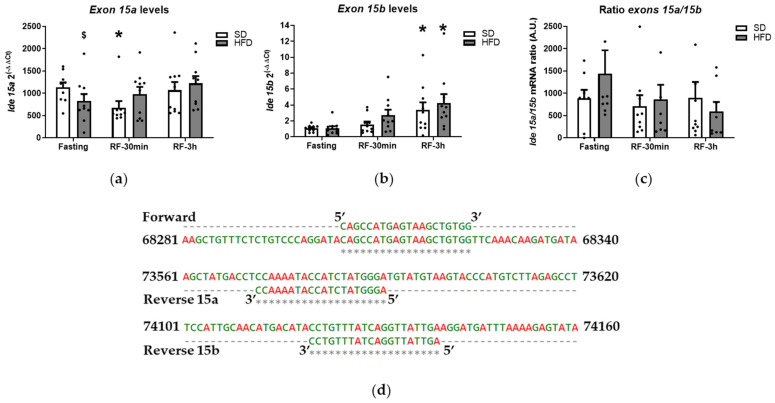
Effects of feeding conditions on levels exons 15a and 15b of mouse *Ide*. (**a**) Levels of 15a *Ide* mRNA in the liver. (**b**) Levels of 15a *Ide* mRNA in the liver. (**c**) Ratio 15a/15b. Data are the means ± SEM. N = 9–10 mice per condition. * *p* < 0.05 vs. fasting by ANOVA. ^$^ *p* < 0.05 vs. SD by ANOVA. (**d**) Alignment of forward and reverse primers to mouse *Ide* gene sequence (NC_000085.7:c37341664-37246140).

Farris and colleagues presented a novel IDE splice isoform in which the canonical exon 15a is replaced by exon 15b, producing a protein with significantly lower catalytic efficiency [26]. To further investigate the changes in IDE activity in response to refeeding conditions, we determined the abundance of exons 15a and 15b by quantitative PCR in the livers of mice fed an SD or HFD. For the first time, we identified both exons in mice, determining that the expression of the 15a splice isoform is ~1000-fold higher than the 15b isoform (Appendix A, Figure A3). However, the observed increase in hepatic IDE activity during refeeding was neither associated with the reduced expression of the 15b isoform, nor with the augmented expression of the 15a isoform (Appendix A, Figure A3). Taken together, these results suggest that metabolites (NEFA, lactate, and glucose) and insulin levels, but not alternative mRNA splicing, influence hepatic IDE activity in response to refeeding.

### 3.3. Kidney IDE Expression and Activity

**Figure 3:**
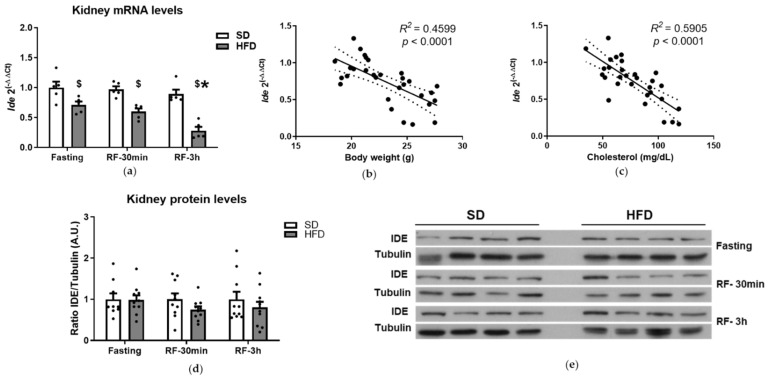
Transcriptional and posttranscriptional regulation of renal IDE. (**a**) Transcriptional regulation of *Ide*. Total mRNA was isolated and quantified from the kidney tissues of mice fed an SD or HFD during fasting or refeeding conditions. Data are the means ± SEM. N = 5 mice per condition. * *p* < 0.05 vs. fasting by ANOVA. ^$^ *p* < 0.05 vs. SD by ANOVA. Regression analyses. Bivariate analyses were performed using data from mice fed an SD and HFD, during fasting and refeeding conditions. (**b**) Correlation between renal *Ide* gene expression and body weight. (**c**) Correlation between renal *Ide* gene expression and plasma cholesterol levels. The *R*^2^ and the statistical significances (*p*) values are indicated in the figure. Regulation of IDE protein levels. IDE protein levels in lysates from kidneys of mice fed an SD or HFD, during fasting or refeeding conditions, were resolved by Western blots and quantified by densitometric analyses using full blots (Appendix A, Figure A2b). Then, data were plotted for each nutritional status and diet (**d**). Data are the means ± SEM. N = 10–11 mice per condition. (**e**) Representative image illustrating renal IDE levels showed in “panel d”.

Li and colleagues have shown the potential significance of renal IDE in the regulation of circulating insulin levels as well as insulin sensitivity [27,28]. To investigate the impact of the fasting-to-refeeding transition on the regulation of IDE levels, we quantified the mRNA and protein levels in the kidneys of mice fed an SD or HFD. As shown in Figure 3a, fasting-to-refeeding did not change *Ide* gene expression in the kidneys of mice fed an SD. However, refeeding decreased its expression in the kidneys of mice fed an HFD (Figure 3a). Interestingly, in mice fed an HFD, we found that plasma NEFA levels directly correlated with *Ide* mRNA levels (*R*^2^ = 0.3577, *p* = 0.018), whereas plasma insulin concentration inversely correlated with *Ide* gene expression levels (*R*^2^ = 0.3527, *p* = 0.019). These results suggest that NEFA and insulin levels might be important in regulating the renal expression of *Ide* during the fasting-to-refeeding transition in mice fed an HFD.

On the other hand, *Ide* gene expression was downregulated in mice fed an HFD compared to SD feeding (Figure 3a). The lowest *Ide* expression levels (~70%) were seen 3 h after refeeding. Additionally, *Ide* gene expression was reduced by ~30% (fasting) and ~40% (refeeding, 30 min) in mice fed an HFD compared to control mice (Figure 3a). Bivariate analysis between *Ide* expression levels and metabolic parameters in mice fed an SD and HFD showed a significant inverse correlation with body weight and cholesterol levels (Figure 3b,c).

Surprisingly, the reductions in *Ide* gene expression in mice fed an HFD was not paralleled by changes in the protein levels (Appendix A, Figure A2b and Figure 3d,e). SD or HFD feeding did not change the IDE protein levels (Appendix A, Figure A2b). Likewise, no differences were found when comparing the IDE protein levels between the mice fed an SD and HFD during the fasting–refeeding transition (Figure 3d,e).

**Figure A4:**
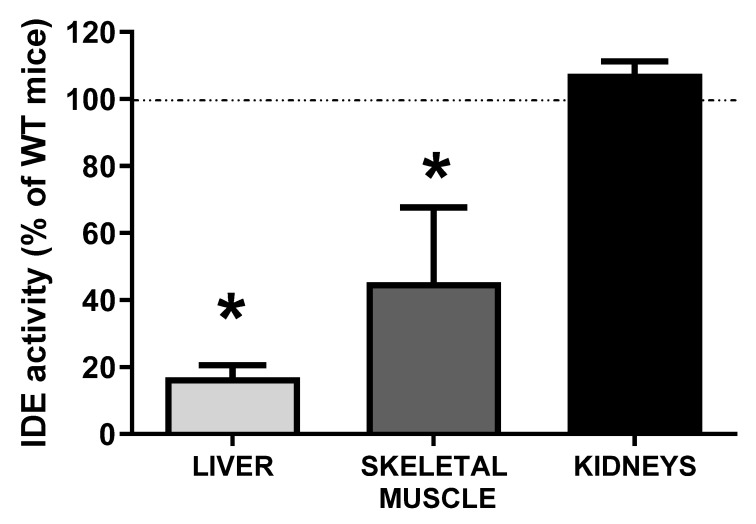
IDE activity in liver, skeletal muscle, and kidneys from WT and IDE-KO mice. Tissue extracts from WT and IDE-KO mice were prepared and assessed for IDE activity. Significance is indicated as relative to the corresponding values of IDE activity in WT mice. Data are the means ± SEM of 3 independent experiments. * *p* < 0.05 vs. WT by ANOVA.

With respect to renal IDE activity levels, we could not reliably assess this parameter using the fluorogenic IDE activity assay, because control experiments showed similar apparent activities (as well as elevated background fluorescence) in kidney extracts from wild-type mice and mice with the pancellular deletion of *Ide* (IDE-KO) (Appendix A, Figure A4). As a plausible explanation, we anticipated that higher levels of fluorescence could come from the autofluorescence of biological components in the tissue, and/or the presence of non-specific proteases that might have affinity for the FRET substrate. Because of these potentially confounding influences, we were unable to obtain IDE activity data for kidney lysates.

### 3.4. Skeletal Muscle IDE Expression and Activity

**Figure 4:**
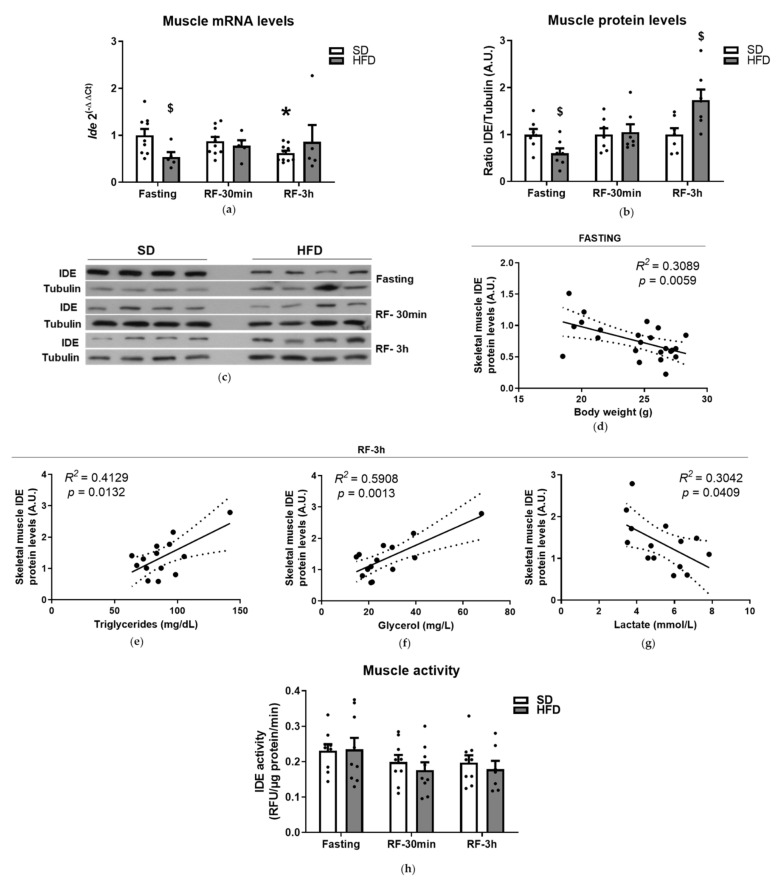
Regulation of IDE mRNA and protein in muscle. (**a**) Regulation of *Ide* gene expression. Total mRNA was isolated and quantified from muscle tissues of mice fed an SD or HFD during fasting or refeeding conditions. Data are the means ± SEM. N = 5–9 mice per condition. * *p* < 0.05 vs. fasting by ANOVA. ^$^ *p* < 0.05 vs. SD by ANOVA. (**b**) Regulation of IDE protein levels. Lysates from muscles of mice fed an SD or HFD, during fasting or refeeding conditions, were resolved by Western blots and IDE was quantified by densitometric analyses using full blots (Appendix A, Figure A2c). Then, data were plotted for each nutritional status and diet. Data are the means ± SEM. N = 10–11 mice per condition. ^$^ *p* < 0.05 vs. SD by ANOVA. (**c**) Representative image illustrating IDE protein levels in muscle showed in “panel b”. Regression analyses. Bivariate analyses were performed using data from mice fed an SD and HFD, during fasting or refeeding conditions. Correlations between IDE protein levels in muscle and body weight (**d**), plasma triglycerides (**e**), plasma glycerol (**f**), and plasma lactate (**g**). The *R*^2^ and statistical significances (*p*) values are indicated in the figure. (**h**) Regulation of IDE activity levels. Effects of the nutritional status on hepatic IDE activity. Lysates from muscles of mice fed an SD or HFD, during fasting or refeeding conditions, were assessed for its activity as described in Section 2. Data are the means ± SEM. N = 9 mice per condition.

Then, we examined the impact of fasting and refeeding in the regulation of *Ide* mRNA levels in the skeletal muscle of mice fed an SD or HFD. As seen in Figure 4a, the fasting-to-refeeding transition reduced the *Ide* gene expression in the muscles of mice fed an SD. The lowest *Ide* expression levels (~70% of fasted controls) were seen 3 h after refeeding (Figure 4a). No correlations were found between muscle *Ide* expression levels and metabolic parameters in mice fed an SD (Appendix A, Table A3). Finally, the fasting-to-refeeding transition did not significantly impact *Ide* mRNA levels in the muscle tissue from mice fed an HFD (Figure 4a). On the other hand, HFD significantly reduced fasting *Ide* gene expression levels by ~40% in comparison to an SD (Figure 4a). However, bivariate analysis did not show any correlation between the muscle *Ide* expression levels and the metabolic parameters of mice fed an SD and HFD (Appendix A, Table A3).

Similar to what was observed in kidneys, changes in muscle *Ide* mRNA levels were not associated with variations in muscle IDE protein levels (Appendix A, Figure A2c and Figure 4b,c). Neither fasting nor refeeding changed IDE protein levels significantly in mice fed an SD or HFD (Appendix A, Figure A2c). However, 8 weeks of HFD feeding resulted in lower (~40%) and higher (~70%) muscle IDE protein levels compared to mice fed an SD under fasting and refeeding (3 h), respectively (Figure 4b,c).

To further investigate feeding-related changes to muscle IDE protein levels in mice fed an SD and HFD, we performed bivariate analyses between IDE levels and metabolic parameters. As shown in Figure 4d, under fasting conditions, body weight inversely correlated with muscle IDE levels. On the other hand, under refeeding (3 h) conditions, plasma triglycerides and glycerol levels directly correlated with IDE levels (Figure 4e,f), whereas plasma lactate inversely correlated with IDE levels (Figure 4g). These results indicate that changes in body weight, and metabolites (triglycerides, glycerol and lactate) levels are able to predict muscle IDE levels. Finally, neither the fasting-to-refeeding transition nor the diet (SD or HFD) affected the proteolytic activity of IDE in skeletal muscle (Figure 4h).

### 3.5. Reduced Liver and Muscle IDE Levels Associate with Insulin Resistance

**Figure 5:**
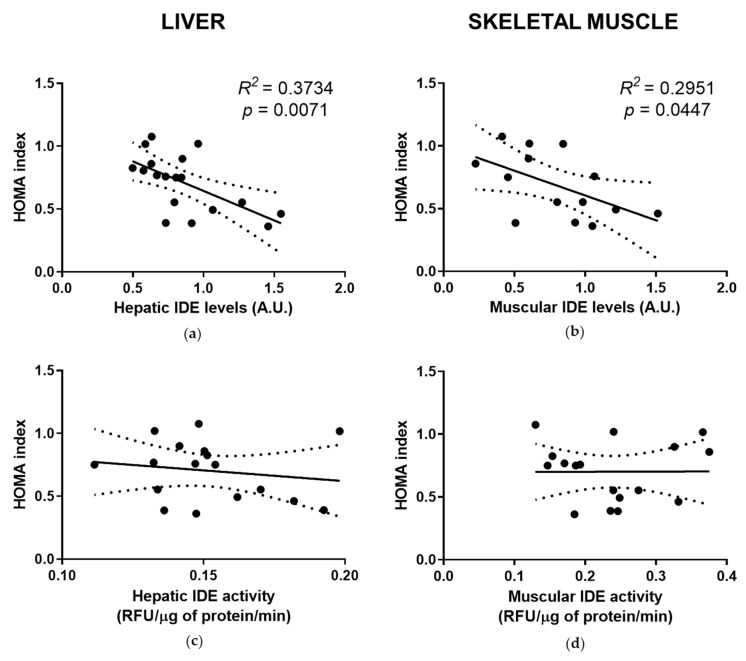
Hepatic and muscle IDE protein levels correlate with a surrogate marker of insulin resistance. IDE protein and activity levels in fasting conditions of mice fed an SD and HFD were assessed as described in Section 2. Bivariate analyses were performed using data from mice fed an SD and HFD under fasting conditions. (**a**) Correlation between hepatic IDE protein levels and HOMA index. (**b**) Correlation between IDE protein levels in muscle and HOMA index. (**c**) Correlation between hepatic IDE activity levels and HOMA index. (**d**) Correlation between muscle IDE activity levels and HOMA index. The *R*^2^ and statistical significances (*p*) values are indicated in the figure.

We showed that HFD feeding led to insulin resistance and glucose intolerance in mice (Appendix A, Figure A1). To further analyze the relationship between IDE and insulin resistance, we performed bivariate analyses between liver and muscle IDE levels in fasting conditions and the HOMA index. As shown in Figure 5a,b, in both tissues there is an inverse correlation between IDE protein levels and the HOMA index. Interestingly, no significant correlations between the IDE activity and HOMA index were found in the liver or skeletal muscle extracts (Figure 5c,d). Taken together, these data suggest that the reduction in IDE levels (~30%) seen during fasting, in the livers and muscle of mice fed an HFD compared to mice fed an SD (Figure 1b and Figure 4b), are associated with the HOMA index, a surrogate assessment of insulin resistance.

### 3.6. Multivariate Analysis

We performed a partial least squares regression (PLSR)-principal component analysis (PCA) to investigate the joint influence of the physiological variables (body weight, metabolites, and hormones) on the IDE mRNA, protein, and activity levels in response to fasting and refeeding.

**Figure 6:**
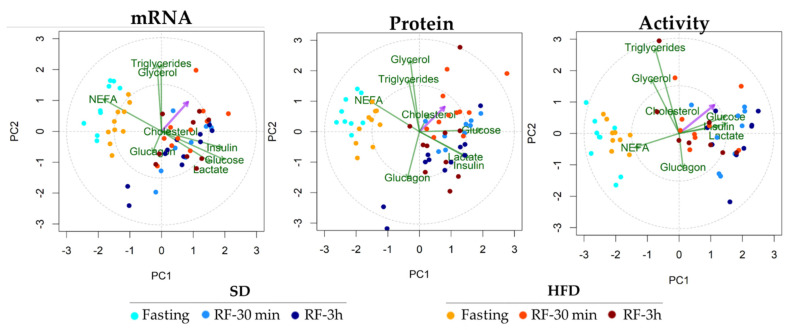
The biplots representation of physiological responses and hepatic IDE regulation in response to nutritional adaptations. The pink arrow shows the variable response (IDE mRNA, protein, or activity levels). The green arrow shows physiological variables (body weight, metabolites, or hormones). Agreement in the direction of pink arrow and green arrows can be interpreted as a graphical proxy for the strength of association between response variable and physiological variables (hormones and metabolites). Dots indicate different nutritional conditions depicted by color codes (see legend in Figure). PC1: principal component 1; PC2: principal component 1.

In the liver, we identified two principal clusters of parameters affected by fasting and refeeding (Figure 6). Under fasting conditions, the NEFA concentration is the main physiological variable found to influence IDE mRNA, protein, and activity levels (Figure 6). NEFA levels correlate inversely with IDE activity, whereas it directly correlates with IDE mRNA and protein levels (Figure 6). In the refeeding states, glucose, lactate, and insulin are the main physiological variables found to correlate with IDE. These variables weakly correlate with mRNA levels, but glucose is a major determinant of protein levels, and all three variables strongly correlate with IDE activity (Figure 6).

**Figure 7:**
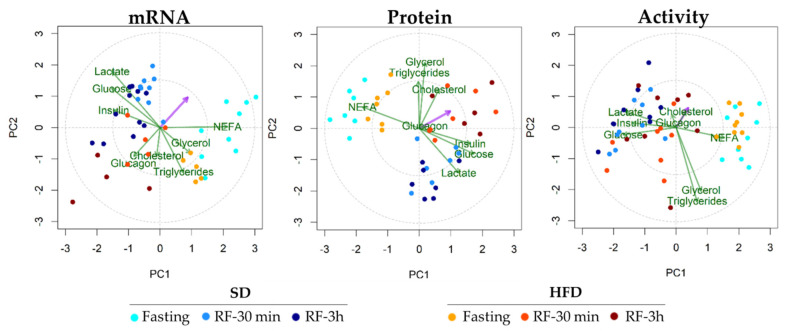
Biplots representation of physiological responses and muscle IDE regulation in response to nutritional adaptations. The pink arrow shows the variable response (IDE mRNA, protein levels, or activity). The green arrow shows physiological variables (body weight, metabolites, or hormones). Agreement in the direction of the pink arrow and green arrows can be interpreted as a graphical proxy for strength of association between response variable and physiological variables (hormones and metabolites). Dots indicate different nutritional conditions depicted by color codes (see legend in Figure). PC1: principal component 1; PC2: principal component 1.

Similar to what we observed in the liver, we identified two principal clusters of metabolic parameters that were influenced by fasting and refeeding in skeletal muscle (Figure 7). Under fasting conditions, NEFA is the main physiological variable associated with the regulation of mRNA, protein, and IDE activity (Figure 7). Under refeeding conditions, lactate, glucose, and insulin associate with changes in mRNA, protein, and IDE activity. Interestingly, these correlations are evident in a standard diet, but disappear in the HFD condition (Figure 7). Finally, no principal clusters were identified between *Ide* mRNA or protein levels and the metabolic parameters of renal tissues (data not shown).

## 4. Discussion

In this study, we undertook a detailed investigation of the effects of fasting and refeeding on the regulation of IDE mRNA, protein and activity levels under conditions of normal and high-fat food consumption. Our study reveals an unexpectedly complex regulation of IDE that is tissue-specific and also depends on circulating levels of hormones and metabolites.

In response to fasting, circulating glucagon levels were lower in mice fed an HFD compared to control mice (Table 1). Different studies have reported disparate results regarding plasma glucagon levels in mice fed an HFD, these being either decreased or elevated in different studies [29,30,31,32,33,34]. Thus, Merino and colleagues reported that mice fed an HFD for 12 weeks exhibited hypoglucagonemia compared to control animals, despite hyperinsulinemia and normoglycemia [32]. Interestingly, these investigators hypothesized that this metabolic phenotype might be an early adaptive response to the eventual progression to T2DM, allowing normoglycemia not only due to the compensatory hyperinsulinemia produced by β-cells, but also by lowering glucagon secretion by α-cells. These findings resembled our current findings, where mice fed an HFD for 8 weeks exhibited lower circulating glucagon levels compared to control mice. We anticipate that longer exposure to HFD feeding (e.g., 24 weeks) would result in hyperglucagonemia, but in our experimental setting (8 weeks), this resulted in hypoglucagonemia.

In the liver, major factors associated with IDE regulation are NEFA (during fasting), and glucose, lactate and insulin (during refeeding). Of note, *Ide* mRNA levels did not always correlate with protein changes, suggesting that one or more additional forms of posttranslational regulatory control may be operative after transcription, such as RNA-binding proteins influencing translation or possibly mechanisms increasing the clearance of IDE protein. Similarly, IDE activity did not always mirror protein levels, suggesting in this case that IDE activity might be modulated by metabolites or possibly interacting proteins. Clearly, these aspects of IDE regulation are poorly understood and deserving of further investigation.

Exposure to increasing amounts of NEFA released from adipose tissue promotes the development of hepatic insulin resistance [35], and some studies have suggested that IDE may be either affected by or involved in this phenomenon. For instance, Hamel and colleagues identified a fatty acid-binding motif within IDE [36] and found that both saturated and unsaturated long-chain free fatty acids (FFAs), and the corresponding acyl-coenzyme A thioesters, inhibited insulin degradation in a non-competitive manner [36]. Furthermore, FFAs decreased insulin degradation in isolated hepatocytes [37] and inhibited the proteolytic activity of IDE released from adipocytes [38]. We recently hypothesized that increasing amounts of saturated FFAs released from adipose tissue via the portal system could inhibit both proteolytic and non-proteolytic functions of IDE, in turn decreasing insulin clearance, whether directly by reducing IDE levels and/or activity or indirectly via effects on insulin receptor internalization and/or recycling. This mechanism may help explain the insulin resistance and hyperinsulinemia seen in obesity [4]. In this study, we found that NEFA levels inversely correlated with hepatic IDE protein levels and activity, these being the most relevant physiological variable found to regulate IDE in the liver. Our findings lend support to the notion that IDE may constitute a mechanistic link between elevated circulating FFAs and hepatic insulin resistance, but further research is needed to confirm this hypothesis.

We previously reported that the genetic deletion of hepatic *Ide* in mice fed an SD or HFD caused insulin resistance or its exacerbation, respectively [16,17]. Unfortunately, this loss-of-function approach cannot discriminate between the effects of the non-proteolytic functions (protein lost) versus proteolytic functions (activity lost). Here, we showed that HFD caused a significant reduction in hepatic IDE levels (~30%) during the fasting state in both liver and skeletal muscle tissues. A remarkable finding of this study is that a surrogate assessment of insulin resistance (HOMA index) inversely correlated with IDE protein levels in liver and muscle tissues, but not with its activity in these tissues, lending support to the notion that the non-proteolytic functions of IDE, rather than proteolytic ones, may be regulating insulin sensitivity in the liver and muscle. These findings raise the question of whether targeting IDE protein levels independently of its activity may be a useful approach for treating insulin resistance. Evidence in the literature suggests that this might be the case, because hepatic IDE overexpression in mice fed an HFD improved insulin sensitivity [16]. Moreover, taurine conjugated bile acid treatment, which augmented hepatic IDE levels without changes in its activity, and improved insulin sensitivity in mice fed an HFD [10].

Early reports identified insulin and glucose as modulators of hepatic IDE activity in vitro. For example, Pivovarova and colleagues showed that insulin stimulated an increase in hepatic IDE activity, but this insulin-mediated effect was abolished in the presence of high-glucose levels in hepatocellular carcinoma HepG2 cells [39]. Although the underlying mechanism(s) by which insulin regulated IDE activity was not established, these investigators showed that the relative proportion of the more proteolytically active 15a splice isoform was increased after insulin treatment, independently of glucose levels [39]. In this study, we found that both insulin and glucose levels directly correlated with hepatic IDE activity in vivo. However, the augmented IDE activity seen during refeeding cannot be explained by changes to *Ide* mRNA splice isoforms, neither to reduced expression of the less proteolytically active 15b isoform, nor to the augmented expression of the more proteolytically active 15a isoform. Our results suggest that the mechanism(s) by which insulin and glucose regulate hepatic IDE activity in mice does not involve alternative mRNA splicing. Nonetheless, our studies highlight the relevance of glucose and insulin as modulators of hepatic IDE activity during refeeding. We postulate that augmented hepatic IDE activity in response to glucose and insulin could serve as a mechanism that preserves hepatocytes from being exposed to high levels of portal insulin, resulting in the downregulation of the insulin receptor and appearance of insulin resistance.

In addition to glucose and insulin, we unexpectedly found that lactate levels directly correlated with hepatic IDE activity and protein levels. Even more interestingly, we established a cause–effect relationship between lactate and the regulation of hepatic IDE activity. In a dose-dependent manner, lactate levels in lysates from livers of mice augmented IDE activity. Lactate’s effect on IDE seems to be attributable not to a direct interaction between IDE and lactate, but to an indirect mechanism most likely involving metabolites and/or other factors related to intracellular lactate metabolism. To the best of our knowledge, this is the first evidence for the lactate-mediated regulation of IDE activity. In any case, further research is warranted to decipher molecular mechanism(s) by which lactate regulates hepatic IDE activity.

In humans, skeletal muscle is a major contributor of circulating lactate, contributing ~40% to total lactate levels in the postabsorptive state [40,41]. Lactate is produced from glucose through glycolysis and the conversion of pyruvate by lactate dehydrogenase [42]. Because lactate production depends on insulin-stimulated glucose uptake, this metabolic pathway is regulated by insulin’s action in skeletal muscle [43]. After its synthesis in muscle, rather than being oxidized there, lactate is released into the systemic circulation, and primarily taken up by the liver [41,44,45], where lactate is used as a gluconeogenic substrate for hepatic glucose production [46]. Interestingly, skeletal muscle exhibits a high capacity to switch from fat to glucose oxidation in the fasting-to-postprandial transition, a term coined “metabolic flexibility” [47,48,49,50]. After a glucose load, glucose is quickly extracted from circulation (15–30 min), allowing its oxidation rather than NEFA oxidation, which is the energy source used during the preceding fast [51,52,53]. In this connection, it is reasonable to hypothesize that lactate is an inter-organ substrate which is produced in muscle and regulates hepatic IDE activity in response to skeletal muscle insulin sensitivity. Our data are consistent with the notion that under conditions of normal insulin sensitivity, such as standard diet feeding, insulin-stimulated glucose uptake is increased in skeletal muscle, allowing a quick switch from fat to glucose oxidation in the fasting-to-postprandial transition. When glycogen stores are replete in muscle, glucose is oxidized through glycolysis, thus raising circulating levels of lactate, which in turn may activate hepatic IDE. Conversely, under insulin-resistant conditions, such as those caused by high-caloric intake, insulin-stimulated glucose uptake is reduced, lowering the rate of lactate production, and so leading to reduced hepatic IDE activity. Through this hypothetical mechanism, the loss of metabolic flexibility in skeletal muscle could be translated into reduced hepatic IDE activation through circulating levels of lactate.

**Figure A5:**
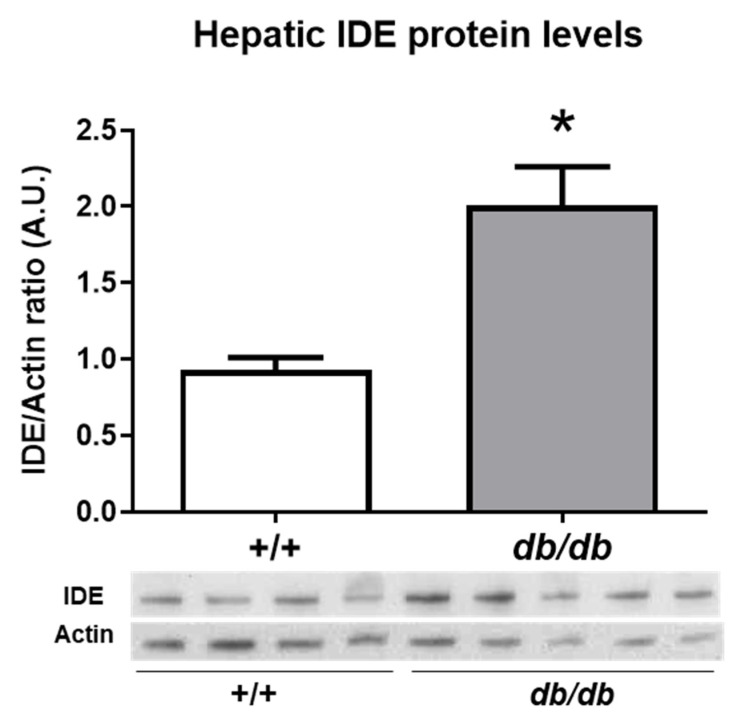
Hepatic IDE protein levels in obese mice. Tissue extracts from the liver of male +/+ and *db*/*db* mice were prepared and analyzed for IDE protein levels. Upper panel: densitometric analyses of IDE. The y axis represents the ratio of IDE versus actin in arbitrary units. Lower panel: representative Western blots depicting IDE. Data are the means ± SEM. N = 9–10 mice per condition. * *p* < 0.05 vs. +/+ by Student’s *t*-test.

Previous studies of fasting and refeeding on hepatic IDE levels in rodent models have yielded contradictory results that are difficult to interpret due to high variability in the length and composition of diets, strains, and sex of animals. Nonetheless, the majority of the studies conducted in mice show that high-caloric feeding for 8–16 weeks results in reduced hepatic IDE levels [6,8,10,11]. We observed similar findings in mice fed an HFD under fasting conditions. Interestingly, longer exposure to a high-caloric intake (6 months) results in higher hepatic IDE levels [7], suggesting that the modulation of IDE protein levels in response to diet is time-dependent. In this regard, we analyzed hepatic IDE levels in a mouse model of chronic obesity (*db*/*db* mice) and found that hepatic IDE levels are higher in *db*/*db* mice compared to the controls (Appendix A, Figure A5). Interestingly, short-term caloric restriction in *db*/*db* mice resulted in lower hepatic IDE levels [54]. Further research is warranted to validate the hypothesis that the upregulation of hepatic IDE protein levels is a mechanism of adaptation to chronic exposure to nutrients.

Skeletal muscle has a key role in glucose homeostasis, accounting for the majority of postprandial glucose disposal under insulin-stimulated conditions. Upon the insulin stimulation of myocytes, glucose has two major fates: glycolysis or glycogen synthesis (the principal pathway in humans) [55,56,57].

Similar to what we observed in the liver, major factors associated with IDE regulation in skeletal muscle are NEFA (during fasting), and glucose, lactate, and insulin (during refeeding) under SD feeding. In contrast to the liver, the transcriptional regulation of *Ide* expression corresponded to changes in protein levels in the fast state, although in both liver and muscle, fasting was associated with reduced IDE protein. Conversely, changes in activity did not mirror changes in protein levels, suggesting that *trans*-acting factors—such as high-abundance metabolites or possibly IDE-interacting proteins—could regulate IDE activity. Given this, future studies on IDE should take into account possible differential effects on IDE mRNA, protein and/or activity levels before reaching firm conclusions.

In summary, this study reveals that the effects of fasting and refeeding and SD versus HFD on IDE in mice are more complex than previously expected. In the liver, protein and activity levels are differentially regulated under fasting and refeeding conditions, whereas in skeletal muscle, IDE protein levels, but not their activity, participate in the fasting-to-postprandial transition. On the other hand, renal IDE to be appeared relatively unaffected by fasting or refeeding. Changes in circulating levels of insulin, glucose, NEFA, and lactate might be principal determinants in regulating IDE in liver and muscle tissues. Finally, IDE protein levels in liver and muscle tissues, but not its activity, strongly correlate with a surrogate assessment of insulin resistance. Collectively, these findings reinforce the idea that IDE could represent a viable pharmacological target for the treatment of insulin resistance.

## Figures and Tables

**Table 1:** Metabolic responses to fasting or refeeding in mice fed an SD or HFD.

	SD			HFD		
	Fasting	Refeeding 30-min.	Refeeding 3-h	Fasting	Refeeding 30-min.	Refeeding 3-h
**Animals (n)**	9	10	10	10	11	11
**Animal Weight (g)**	19.94 ± 0.33	21.37 ± 0.43	21.41 ± 0.32	25.85 ± 0.34 ^$^	25.73 ± 0.55 ^$^	25.46 ± 0.37 ^$^
**CI (kcal/kg)**	―	96.42 ± 4.02 ^#^	258.25 ± 15.55 ^#^	―	87.77 ± 10.20 ^#^	192.11 ± 16.70 ^#^
**CI rate (kcal/kg/min)**	―	3.21 ± 0.13 ^#^	1.43 ± 0.09 ^#^	―	2.93 ± 0.34 ^#^	1.07 ± 0.09 ^#^
**Glucose (mg/dL)**	96.33 ± 3.87	214.80 ± 8.17 *^#^	173.40 ± 6.30 *^#^	111.60 ± 5.26	218.82 ± 8.97 *	174.55 ± 4.89 *
**Insulin (ng/mL)**	0.09 ± 0.01	1.13 ± 0.12 *	1.09 ± 0.17 *	0.13 ± 0.00 ^$^	0.92 ± 0.15 *	1.55 ± 0.25 *^$^
**Glucagon (pmol/L)**	33.51 ± 8.75	12.38 ± 1.27	46.97 ± 19.37	8.02 ± 1.07 ^$^	13.33 ± 1.94	30.67 ± 12.12 *
**Cholesterol (mg/dL)**	61.00 ± 5.06	69.45 ± 3.76	59.91 ± 5.38	93.77 ± 3.50	91.79 ± 6.52	106.18 ±5.17 ^$^
**Triglycerides (mg/dL)**	88.12 ± 7.40	71.63 ± 5.40	69.40 ± 5.50	94.59 ± 3.41	89.31 ± 5.33	91.74 ± 5.63
**NEFA (mmol/L)**	1.75 ± 0.12	0.65 ± 0.03	0.41 ± 0.05 *	1.00 ± 0.03	0.37 ± 0.02 *	0.30 ± 0.02
**Glycerol (mg/L)**	35.57 ± 1.43	26.06 ± 0.88	17.72 ± 1.26 *	37.62 ± 2.36	38.96 ± 2.10 ^$^	35.14 ± 3.54 ^$^
**Lactate (mmol/L)**	2.62 ± 0.33	6.23 ± 0.28 *	6.52 ± 0.24 *	1.98 ± 0.08	4.15 ± 0.33 ^$^	4.12 ± 0.19 ^$^

Data are the means ± SEM. * *p* < 0.05 vs. fasting by two-way ANOVA; ^$^ *p* < 0.05 vs. SD; ^#^ *p* < 0.05 vs. refeeding by two-way ANOVA. CI: Caloric Intake.

## Data Availability

The guarantors for the content of the article are I.C.-C. and G.P. The data presented in this study are available within the article and upon request from the corresponding author.

## Appendix A

**Table A1:** *R*^2^ values for hepatic *Ide* mRNA levels and metabolic parameters in mice fed an HFD. n.s., not statistically significant.

Variable	Body Weight (g)	Glucose (mg/dL)	NEFA (mmol/L)	Cholesterol (mg/dL)	Triglycerides (mg/dL)	Glycerol (mg/L)	Lactate (mmol/L)	Insulin (ng/mL)	Glucagon (pmol/L)
*Ide* mRNA levels (HFD)	0.0393	0.0685	0.1250	0.0662	0.0069	0.0010	0.1422	0.1600	0.1251
*p* values	n.s.	n.s.	n.s.	n.s.	n.s.	n.s.	0.0399	0.031	n.s.

**Table A2:** *R*^2^ values for hepatic IDE protein levels and metabolic parameters in mice fed an HFD. n.s., not statistically significant.

Variables	Body Weight (g)	Glucose (mg/dL)	NEFA (mmol/L)	Cholesterol (mg/dL)	Triglycerides (mg/dL)	Glycerol (mg/L)	Lactate (mmol/L)	Insulin (ng/mL)	Glucagon (pmol/L)
IDE protein levels fasting (HFD)	0.0382	0.0405	0.3840	0.0372	0.2374	0.0428	0.02958	0.1451	0.0737
*p* values	n.s.	n.s.	n.s.	n.s.	n.s.	n.s.	n.s.	n.s.	n.s.

**Table A3:** *R*^2^ values for muscle *Ide* mRNA levels and metabolic parameters in mice. n.s., not statistically significant.

Variable	Body Weight (g)	Glucose (mg/dL)	NEFA (mmol/L)	Cholesterol (mg/dL)	Triglycerides (mg/dL)	Glycerol (mg/L)	Lactate (mmol/L)	Insulin (ng/mL)	Glucagon (pmol/L)
*Ide* mRNA levels (SD)	0.009	0.003	0.1703	0.023	0.006	0.08	0.1496	0.069	0.1022
*Ide* mRNA levels (HFD)	0.177	0. 221	0.052	0.1068	0.017	0.033	0.26	0.044	0.044
*Ide* mRNA levels (SD and HFD)	0.268	0.049	0.242	0.115	0.021	0.085	0.020	0.187	0.266
*p* values	n.s.	n.s.	0.01	n.s.	n.s.	n.s.	0.03	n.s.	n.s.
	n.s.	n.s.	n.s.	n.s.	n.s.	n.s.	n.s.	n.s.	n.s.
	n.s.	n.s.	n.s.	n.s.	n.s.	n.s.	n.s.	n.s.	n.s.
